# A Leopard Cub ( *Panthera pardus kotiya* ) Adopted by Kin and Non‐Kin Leopardesses Consecutively

**DOI:** 10.1002/ece3.70952

**Published:** 2025-02-08

**Authors:** Dushyantha Silva, Milinda Wattegedera, Mevan Piyasena, Raveendra Siriwardene, Sasindu Hewage, Meegasthanne Gamaralalage Chandana Sooriyabandara, Marasinghe Sumanasirige Lesly Ranjan Pushpakumara Marasinghe, Rajapakse M. R. Nilanthi, Prasantha Wimaladasa, Gotabhaya Ranasinghe, Kaveesha Perera

**Affiliations:** ^1^ Research and Data Gathering Team Yala Leopard Center Hambantota Sri Lanka; ^2^ Department of Wildlife Conservation of Sri Lanka Funding Battaramulla Sri Lanka; ^3^ Institute of Cardiology National Hospital of Sri Lanka Colombo Sri Lanka

## Abstract

The study examines alloparental care and adoption in the Sri Lankan leopard population at Yala National Park, Sri Lanka. Using the multi‐point leopard identification method, it documents a remarkable instance: a leopard cub initially adopted by its maternal aunt and later by an unrelated female with no prior connection to the cub. The cub had a sibling litter mate, who was not seen in the park after the initial adoption ended by the maternal aunt. We consider this behavior noteworthy in especially solitary animal species such as leopards. Our study was conducted for a period of 2 years and 5 months (from February 2021 to July 2023). Our observations highlight the dynamics of these adoptions and the behaviors exhibited. Altruistic acts, such as feeding, brushing, and shielding the cubs, were observed. These inspections challenge the conventional knowledge regarding leopard behaviors. Notably, the adoption of the cub by unrelated females, despite the absence of genetic ties, exemplifies a form of mutually beneficial reciprocal altruism, offering advantages to both parties. The following study explains leopard adoptions using evolutionary theories like kin altruism and reciprocal altruism. It suggests factors such as genetic relatedness, mutual dependency, and possible misidentification influenced the act of adoption. These rare altruistic acts benefit the leopard population. Our study opposes traditional concepts of solitary Sri Lankan leopards. Altruistic behaviors, influenced by genetic relatedness and reciprocal benefits, emphasize social dynamics in predator populations. These findings enhance the understanding of evolutionary mechanisms and cooperative behaviors in maintaining population fitness in the Sri Lankan leopard population.

## Graphical abstract

This study examines alloparental care and adoption in the Sri Lankan leopard population at Yala National Park, documenting a case where a cub was adopted by both a maternal aunt and an unrelated female. These findings challenge traditional views of solitary leopards, suggesting that altruistic behaviors, influenced by genetic relatedness and reciprocal benefits, play a crucial role in population fitness.

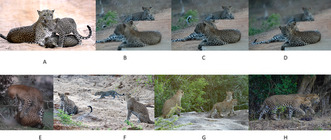

## 1 Introduction

The *Panthera pardus kotiya* , commonly known as Sri Lankan leopard, is a subspecies of the *Panthera pardus* family (Miththapala et al. 1996). It is the apex predator of Sri Lanka and lives in the montane, submontane, tropical rain, monsoonal, dry, evergreen, and arid zone scrub forests of Sri Lanka (Wattegedera et al. 2022). The leopards of the Yala National Park are being identified using the “Multi‐Point Leopard Identification Method” (Wattegedera et al. 2022) on an on‐going basis since the year 2013. Yala has a density of one per 0.54 km^2^ of leopard population density (Wattegedera et al. 2022).

Altruistic behavior through alloparenting and adoption was recorded in 120 species of mammals and 150 species of birds (Riedman 1982). An act of altruism benefits the other at a cost to the self of the performer (Darlington 1978). Alloparenting is an extended form of altruism (Darlington 1978). Alloparenting is to feed, groom, assist young in stressful situations and babysit another individual's offspring (Jennions and Macdonald 1994). Behavior of an older female who is not a parent, caring for a younger is also termed as aunting in primate behavior (Hrdy 1976). Riedman termed adoption as an extension of alloparenting by providing foster parental care in the absence of the actual parents (Riedman 1982). Alloparenting derives indirect fitness benefits to all alloparents (Bergmüller et al. 2007). Indirect fitness to alloparents can be in the form of hunting together by both adopter and adoptee and babysitting while the adopter is away.

Altruism and adoption has been displayed by many mammals and birds: including pumas ( *Puma concolor* ), (Bartnick et al. 2013) lions ( *Panthera leo* ), (Smith and Kok 2006) red squirrels ( *Tamiasciurus hudsonicus* ), (Gorrell et al. 2010) primates (Hrdy 1976), African wild cats (*Felis silvestris libyca*), (Crowell‐Davis 2007) feral and domestic cats ( *Felis catus* ), (Crowell‐Davis et al. 2004) cheetahs ( *Acinonyx jubatus* ), (Caro 1994) and a single instance of a leopard ( *Panthera pardus* ). (Balme et al. 2012; Hunter et al. 2013) For example, a female puma adopted two orphaned juvenile males, whereas in lions, a male cared for his female sibling's cubs to support her. In feral cat colonies, adult females cooperate in caring for the kittens, and among cheetahs, sibling brothers live together and cooperate in their activities. They are polyestrous and are known to be hostile toward conspecifics but more tolerant toward their daughters (Balme et al. 2012; Owen et al. 2010).

In this study, we recorded an instance where two male leopard cubs were adopted by their maternal aunt for two and a half months before she ended the adoption. Subsequently, one of the cubs went was not seen in the area and the other one of those cubs was adopted by another female leopard who has no relations within the nearest two generations of the cub. The new adopter provided for a litter of her own twin cubs, a male and female of around 3 months of age at the time of the adoption.

## 2 Methodology

The study is done using digital single‐lens reflex (DSLR) and mirrorless cameras to identify individual leopards using the capture recapture method basing the multi‐point leopard identification method. The multi‐point leopard identification method considers the changes that occur to the form and shape of the spots and rosettes of a leopard and recommends that nine out of the 16 segregations of a leopard's skin coat are matched when identifying a leopard. Photography and observations have been used to document the behavior of identified individual leopards. The identification database of the Yala National Park was used to identify the photographed and observed individuals. The data gathering was done by 103 days of visits to the national park spending 6 h from February 2021 to July 2023.

## 3 Observation

**FIGURE 1:**
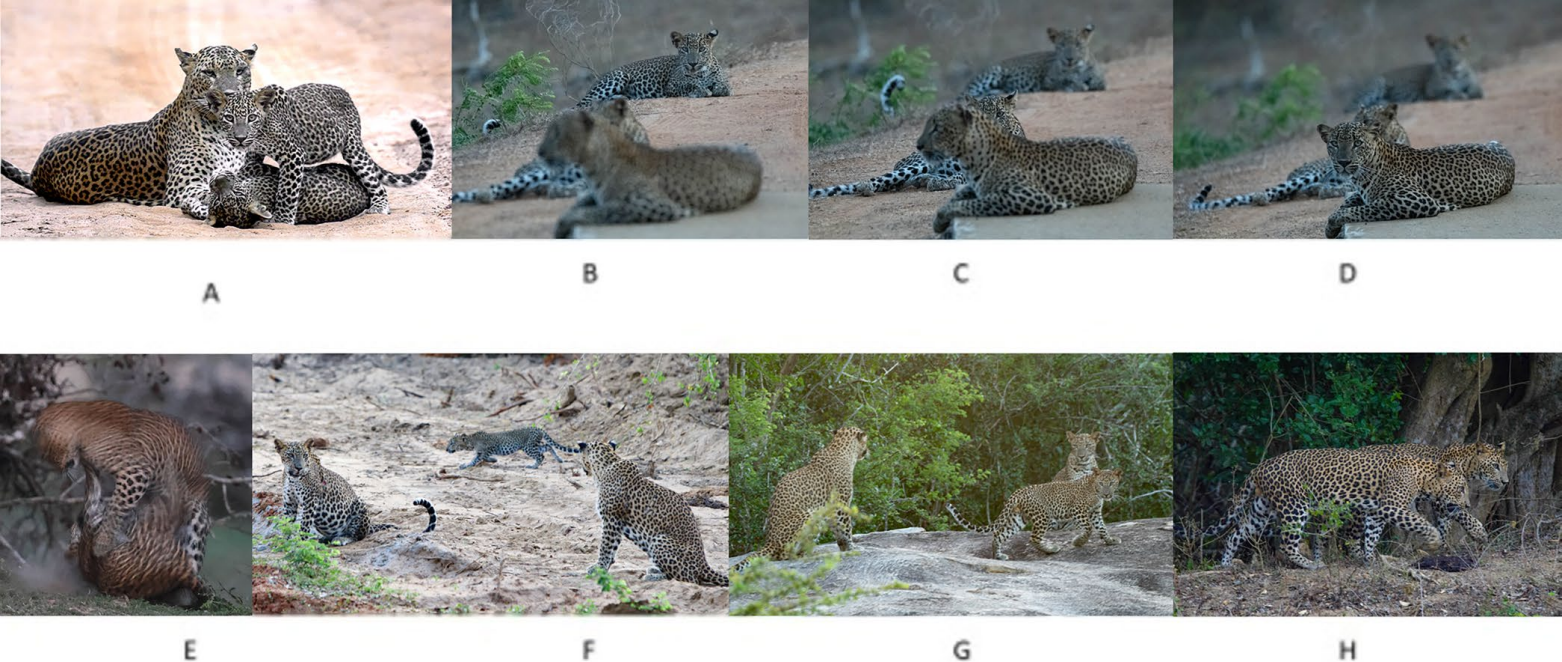
Template I (Photo A‐YF 2 parenting her cubs, YM 67 and YM 68; Photo B/C/D‐ YM 67 and YM 68 together with their maternal aunt YF 46; Photo E‐mild aggression between YF 46 and YM 67 trying to share a deer carcass; Photo F/G/H‐YM 67 was observed always together with YF 15 and her cubs YM 70 and YF 68 until YF 15 separated from her cubs).

**FIGURE 2:**
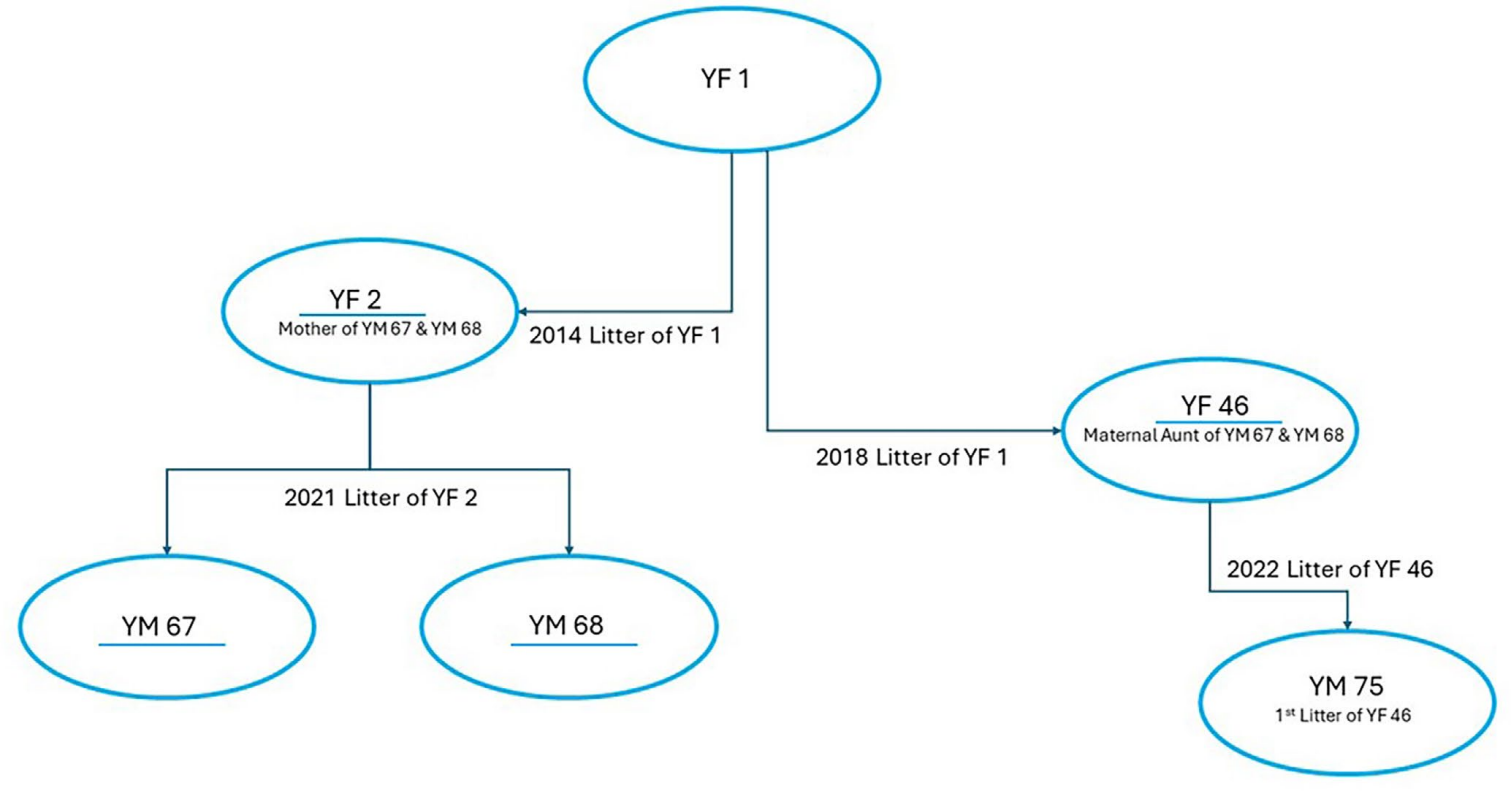
Documented maternal family tree of YM 67 and YM 68 (YF 2, YF 46, YM 67, and YM 68 are underlined in blue).

We observed two male cubs, YM 67 and YM 68, first recorded them in February 2021, as cubs of around 2 months of age. They were seen being parented by their mother, YF 2 at the time of observation. The mother and cubs were observed regularly until July 2021 (Figure 1A). We observed that there were no sightings of the mother, YF 2, thereafter, though there were brief sightings of the cubs. In August 2021, there was an observation of the cubs, YM 67 and YM 68 together with their maternal aunt YF 46, who is not a litter mate but an offspring of the previous litter of the mother of YF 1 (Figure 1B–D). The maternal family tree of cubs YM 67 and YM 68 shown as follows (Figure 2).

As per our observations, female YF 46 was born in the year 2018 and had no litter and no previous litters or cubs at that time. Female YF 46 was around 3 years at the time she adopted the cubs. The cubs and female YF 46 were seen together 10 times thereafter. They were seen drinking water, walking, and sharing kills together. There was also mild hostility when the kills were shared between them as female YF 46 would first eat the kill and then allow the cubs to eat the rest after she fed on it. Figure 1E,F, taken in September 2021, shows such an instance observed of mild aggression which lasted for just 5 s when YM 67 and YM 68 tried to share a carcass of a spotted deer (*Axis axis Ceylonesis*) when YF 46 was feeding. However, she allowed them to feed on the rest of the carcass after she had fed (Figure 1E).

There were no observations of female YF 46 together with cub YM 67 and cub YM 68 from October 2021. YM 68 was observed alone on three occasions between October and February 2022. There were no sightings of cub YM 68 since then.

In October 2021, we observed female YF 15 with a twin litter of approximately 3‐month‐old cubs. The cubs were recorded as one male (YM 70) and one female (YF 68). Female YF 15 was estimated to be around 9 years old. In total, she had three previous litters which produced six cubs of which she successfully raised only 50% of the cubs to separation. Her immediate previous litter in 2020 was a set of three cubs who did not survive up to the age of 6 months. In October 2021, we observed cub YM 67 with female YF 15 and her own cubs (Figure 1F). YM 67 was around 10 months old when he was first sighted with female YF 15. Cub YM 67 has no known maternal relationship with female YF 15. There were no observed interactions between cub YM 67's maternal grandmother's offsprings recorded from the inception of our research in 2013. Cub YM 67, female YF 15 and her two cubs, cub YM 70 and cub YF 68 was observed always together until the separation of female YF 15 from her own cubs in 2023 (Figure 1G).

Cub YM 67 was observed with cub YM 70 even after separation from female YF 15 in February 2022. There was no observed hostility between female YF 15 or her cubs, with cub YM 67 at any point. They were seen walking, scent marking, sharing kills, going after prey, playing, and resting together. Figure 1H shows cub YM 67 and cub YM 70 together in April 2022.

We observed female YF 46 with her own 3‐month‐old male cub identified as YM 75, in July 2022, which is 9 months after she ended the adoption of cubs YM 67 and YM 68.

## 4 Discussion

### 4.1 Adoption of Cubs YM 67 and YM 68 by Female YF 46

Kin altruism is when an individual makes personal sacrifices for the genetical gains (Darlington 1978). A nephew shares one fourth of the genetics in a sexually producing Mendelian population (Darlington 1978). Cubs YM 67 and YM 68 are both nephews of female YF 46. Kin altruism increases the probability of carrying genes (Hamilton 1963). Altruism care given to kin can enhance inclusive fitness. Inclusive fitness is defined as the total effect of the carers own genetical fitness and the effect of its action can affect the fitness of the kin (Hamilton 1963). Inclusive fitness can be achieved through one's own reproduction or by reproduction of relatives of the same genes (Moore and Michel 1998). Female YF 46 adopted her two nephews which suggests kin altruism. The altruism care given to them had a cost to her in terms of food as she must take extra effort to provide for the two male leopard cubs, hence it may be the reason of mild hostility in sharing kills. Female YF 46 was observed interacting together without any hostility in previous instances with female YF 2 both before and after the birth of cubs YM 67 and YM 68. They shared the same home range as female YF 1, their mother. The altruistic care shown to cubs YM 67 and YM 68 could have reciprocally benefited female YF 46 in terms of gaining parenting skills in raising her own offspring in addition to the above‐mentioned reason. The altruistic care shown by female YF 46 toward the cubs quantify the Hamilton's theory of Evolution of altruism, as it mentions that altruism is shown among close relatives (Hamilton 1963).

Female YF 46 was observed with a male cub of an estimated age of 3 months in July 2022, which is 7 months after she ended the Altruistic care to cubs YM 67 and YM 68. A leopards gestation period is generally between 90 and 106 days (Furstenburg 2010).

The reason for female YF 46 to end the altruistic care may be due to two reasons which are that she could have reduced her fecundity by prolonging the care given to the cubs (Atkinson et al. 1996), and the cost tradeoff she would have encountered to share resources, as she would have had to prepare herself for her own pregnancy. This was described as Pavlov strategy as a “win stay–lose shift” policy, where animals will shift to a higher payoff from a lower payoff situation (Clements and Stephens 1995). This can be termed as a negative pseudo‐reciprocity where one would terminate altruism with the intention of avoiding fitness losses due to the actions of the other (Bergmüller et al. 2007).

### 4.2 Adoption of Cub YM 67 by YF 15

Adoption can be defined as advanced alloparenting (Riedman 1982), which embody the essential characteristics of parenting by a nonparent. Adoption was explained in studies of Goslings as when foreign young join a brood and the parents of that brood give inclusive parental care to the new nonfamily members (Eadie et al. 1988). One of the cubs, YM 67 was observed with female YF 15 on 23 October 2021 within her home range.

Hamilton stated that altruism was performed among a set of close relatives or due to misidentification of an offspring (Hamilton 1963). Trivers stated that altruism can be performed by nonkin (Trivers 1971) through reciprocal altruism. Reciprocal altruism is an act of altruism done in expecting an altruistic return by kin or nonkin. Female YF 15 was not related to cub YM 67 for two generations preceding him. She was never observed to have any interactions with female YF 1 (maternal grandmother of cub YM 67), female YF 2 (mother of cub YM 67), and female YF 46 (aunt of cub YM 67). The adoption of cub YM 67 can be termed as a nonkin reciprocal altruism.

Trivers stated many benefits that individuals who adopt can gain through reciprocal altruism, out of which mutual dependency is the probable benefit in this case (Trivers 1971). Mutual dependency can be defined as the benefits that are reciprocally gained through depending on each other's interactions when they operate as a group. We observed three instances where female YF 15 gained advantages by using cub YM 67 (adoptee), who was in subadulthood, to stalk behind a herd of deer creating chaotic confusion, and she ambushed the herd from the other side and killed one of them. Both leopards reciprocally benefited here as female YF 15 gained continuous quick successful hunts for her and her own offsprings, whereas cub YM 67 benefited by his share of the meal and the parental like training of hunting techniques. Avital et al. (1998) stated that the adoptee acquires adoptive parenting styles from the adopter, which will evolve genetically over time. We also observed four instances which where female YF 15's cubs both YM 70 and YF 68 or either of them, were together with cub YM 67, when female YF 15 was seen in other areas of her home range, which amounts to the benefit of mutual dependency of protection, cub YM 67 would also get the reciprocal benefit of protection from other leopards due to the adoption.

Avital et al. (1998) stated of another possible reason that adoption can occur is due to misidentification of one's own offspring and taking responsibility for another's offspring. There could be a remote chance that female YF 15 adopted cub YM 67 by misidentification and assuming it is one of the cubs from her previous litter who disappeared and is presumed to be dead. The previous litter was observed as 3‐month‐old cubs in July 2020, and they are presumed dead as they were not observed after September 2020 and that female YF 15 was seen with a new set of twin cubs in October 2021. A previous study conducted stated that a leopards fecundity is increased by loss of cubs (Balme et al. 2013).

## 5 Conclusion

We describe the first case of leopard cub siblings having been afforded alloparental care by a maternal aunt and subsequently, one of those cubs being adopted by another female, not closely related to the cub which at the time had a litter of her own young cubs. This occurred in Yala National Park, Sri Lanka. The leopard population has been continuously monitored by our research team since 2013. Female YF 46 adopted cubs YM 67 and YM 68, her maternal nephews, after their mother: female YF 2, went missing. This kin altruism likely benefited female YF 46 through genetic fitness and the acquisition of parental skills. The adoption supports W.D. Hamilton's theory of altruism. After 2.5 months, YF 46 ended her care for the cubs, and in July 2022, she was seen with her own offspring. This behavior aligns with Pavlov's win‐stay, lose‐shift strategy, and Bergmüller's pseudo negative reciprocal altruism.

## Author Contributions

**Dushyantha Silva:** conceptualization (lead), data curation (lead), formal analysis (lead), investigation (lead), methodology (lead), project administration (lead), resources (lead), supervision (lead), validation (lead), visualization (lead), writing – original draft (lead), writing – review and editing (equal). **Milinda Wattegedera:** conceptualization (equal), data curation (equal), investigation (equal), methodology (equal), resources (equal), validation (equal), visualization (equal), writing – original draft (equal). **Mevan Piyasena:** investigation (equal), resources (equal). **Raveendra Siriwardene:** data curation (equal), visualization (equal). **Sasindu Hewage:** resources (equal). **Meegasthanne Gamaralalage Chandana Sooriyabandara:** investigation (equal). **Marasinghe Sumanasirige Lesly Ranjan Pushpakumara Marasinghe:** investigation (equal). **Rajapakse M. R. Nilanthi:** visualization (equal), writing – review and editing (equal). **Prasantha Wimaladasa:** investigation (equal). **Gotabhaya Ranasinghe:** data curation (equal), resources (equal), visualization (equal), writing – original draft (equal), writing – review and editing (equal). **Kaveesha Perera:** writing – original draft (equal), writing – review and editing (equal).

## Ethics Statement

The research was carried out with permission and under the supervision of the Department of Wildlife Conservation (DWC), permit no. WL/O3/02/78/15. We carried out the research ethically. We have not created disturbances to the animals. The research was noninvasive. No harm was caused to any animals due to the study. We did not use bait and lures in the research.

## Conflicts of Interest

The authors declare no conflicts of interest.

## Data Availability

All supporting figures and list of references used to compile this manuscript were added to Mendeley Data under the citation; Silva, Dushyantha; Wattegedara, Milinda; Piyasena, Mevan; Siriwardene, Raveendra; Ranasinghe, Gotabhaya; Perera, Kaveesha; Hewage, Sasindu; Sooriyabandara, C; Marasinghe, Ranjan; Rajapakse, Nilanthi; Wimaladasa, Prasantha (2024), “A Leopard Cub ( *Panthera pardus kotiya* ) Adopted by Kin and Non‐Kin Leopardesses Consecutively,” Mendeley Data, V1, doi: 10.17632/4gxmb97njy.1.
